# Life-Times

**Published:** 1857-12

**Authors:** 


### LIFETIMES.

| | Years. | | Years. |
|---|---|---|---|
| A MAN has lived . . . | 970 | Porpoise . . . . . | 30 |
| Whale (estimated) . . | 900 | Bear . . . . . . . . | 20 |
| Elephant . . . . . . | 400 | Dog . . . . . . . . | 20 |
| Swan . . . . . . . | 360 | Wolf . . . . . . . . | 20 |
| Tortoise . . . . . . . | 107 | Rhinoceros . . . . . | 20 |
| Eagle . . . . . . . . . | 104 | Cat . . . . . . . . | 20 |
| Camel . . . . . . . | 100 | Fox . . . . . . . . | 16 |
| Raven . . . . . . . | 100 | Cow . . . . . . . . | 15 |
| Lion . . . . . . . . . | 70 | Sheep . . . . . . . | 10 |
| Horse . . . . . . . | 62 | Squirrel . . . . . . . | 8 |
| Pig . . . . . . . . . | 30 | Rabbit . . . . . . . | 8 |
| Dolphin . . . . . . | 30 | Good Resolutions, | an hour. |

